# BonoboFlow: viral genome assembly and haplotype reconstruction from nanopore reads

**DOI:** 10.1093/bioadv/vbaf115

**Published:** 2025-05-13

**Authors:** Christian Ndekezi, Drake Byamukama, Frank Kato, Denis Omara, Angella Nakyanzi, Fortunate Natwijuka, Susan Mugaba, Alfred Ssekagiri, Nicholas Bbosa, Obondo James Sande, Magambo Phillip Kimuda, Denis K Byarugaba, Anne Kapaata, Jyoti Sutar, Jayanta Bhattacharya, Pontiano Kaleebu, Sheila N Balinda

**Affiliations:** Medical Research Council/Uganda Virus Research Institute & London School of Hygiene and Tropical Medicine (MRC), Entebbe, P.O. Box 49, Uganda; College of Health Sciences, department of Immunology and Molecular Biology, Makerere University, Kampala, P.O. Box 7062, Uganda; Uganda Virus Research Institute, Entebbe, P.O. Box 49, Uganda; Medical Research Council/Uganda Virus Research Institute & London School of Hygiene and Tropical Medicine (MRC), Entebbe, P.O. Box 49, Uganda; Medical Research Council/Uganda Virus Research Institute & London School of Hygiene and Tropical Medicine (MRC), Entebbe, P.O. Box 49, Uganda; College of Health Sciences, department of Immunology and Molecular Biology, Makerere University, Kampala, P.O. Box 7062, Uganda; Medical Research Council/Uganda Virus Research Institute & London School of Hygiene and Tropical Medicine (MRC), Entebbe, P.O. Box 49, Uganda; College of Health Sciences, department of Immunology and Molecular Biology, Makerere University, Kampala, P.O. Box 7062, Uganda; Uganda Virus Research Institute, Entebbe, P.O. Box 49, Uganda; Uganda Virus Research Institute, Entebbe, P.O. Box 49, Uganda; Medical Research Council/Uganda Virus Research Institute & London School of Hygiene and Tropical Medicine (MRC), Entebbe, P.O. Box 49, Uganda; College of Health Sciences, department of Immunology and Molecular Biology, Makerere University, Kampala, P.O. Box 7062, Uganda; Medical Research Council/Uganda Virus Research Institute & London School of Hygiene and Tropical Medicine (MRC), Entebbe, P.O. Box 49, Uganda; Uganda Virus Research Institute, Entebbe, P.O. Box 49, Uganda; Medical Research Council/Uganda Virus Research Institute & London School of Hygiene and Tropical Medicine (MRC), Entebbe, P.O. Box 49, Uganda; Uganda Virus Research Institute, Entebbe, P.O. Box 49, Uganda; College of Health Sciences, department of Immunology and Molecular Biology, Makerere University, Kampala, P.O. Box 7062, Uganda; College of Veterinary Medicine Animal Resources and Biosecurity, Department of Biomedical Laboratory Technology and Molecular Biology (BLT), Makerere University, Kampala, P.O. Box 7062, Uganda; College of Veterinary Medicine Animal Resources and Biosecurity, Department of Biomedical Laboratory Technology and Molecular Biology (BLT), Makerere University, Kampala, P.O. Box 7062, Uganda; Medical Research Council/Uganda Virus Research Institute & London School of Hygiene and Tropical Medicine (MRC), Entebbe, P.O. Box 49, Uganda; Antibody Translational Research Program, Center for Virus Research, Vaccines & Therapeutics, BRIC-Translational Health Science & Technology Institute, NCR Biotech Science Cluster, Faridabad, Haryana 121001, India; IAVI, Gurugram, Haryana-122002, India & New York, NY 10004, USA; Antibody Translational Research Program, Center for Virus Research, Vaccines & Therapeutics, BRIC-Translational Health Science & Technology Institute, NCR Biotech Science Cluster, Faridabad, Haryana 121001, India; Molecular and Translational Virology Unit, Center for Virus Research, Vaccines & Therapeutics, BRIC-Translational Health Science & Technology Institute, NCR Biotech Science Cluster, Faridabad, Haryana 121001, India; CEPI Central Laboratory Network (CLN), Bioassay Laboratory, BRIC-Translational Health Science & Technology Institute, NCR Biotech Science Cluster, Faridabad, Haryana 121001, India; Medical Research Council/Uganda Virus Research Institute & London School of Hygiene and Tropical Medicine (MRC), Entebbe, P.O. Box 49, Uganda; Uganda Virus Research Institute, Entebbe, P.O. Box 49, Uganda; Medical Research Council/Uganda Virus Research Institute & London School of Hygiene and Tropical Medicine (MRC), Entebbe, P.O. Box 49, Uganda; Uganda Virus Research Institute, Entebbe, P.O. Box 49, Uganda

## Abstract

**Summary:**

Viral genome sequencing and analysis are crucial for understanding the diversity and evolution of viruses. Traditional Sanger sequencing is limited by low sequence depth and is labor intensive. Next-Generation Sequencing (NGS) methods, such as Illumina, offer improved sequencing depth and throughput but face challenges with accurate reconstruction of viral genomes due to genome fragmentation. Third-generation sequencing platforms, such as PacBio and Oxford Nanopore Technologies (ONT), generate long reads with high throughput. However, PacBio is constrained by substantial resource requirements, while ONT suffers from inherently high error rates. Moreover, standardized pipelines for ONT sequencing encompassing basecalling to genome assembly remain limited.

**Results:**

Here, we introduce BonoboFlow, a standardized Nextflow pipeline designed to streamline ONT-based viral genome assembly/haplotype reconstruction. BonoboFlow integrates key processing steps, including basecalling, read filtering, chimeric read removal, error correction, draft genome assembly/haplotype reconstruction, and genome polishing. The pipeline accepts raw POD5 or basecalled FASTQ files as input, produces FASTA consensus files as output, and uses a reference genome (in FASTA format) for contaminant read filtering. BonoboFlow’s containerized implementation via Docker and Singularity ensures seamless deployment across diverse computing environments. While BonoboFlow excels in assembling small and medium viral genomes, it showed challenges when reconstructing large viral genomes.

**Availability and implementation:**

BonoboFlow and corresponding containerized images are publicly available at https://github.com/nchis09/BonoboFlow and https://hub.docker.com/r/nchis09/bonobo_image. The test dataset is available at SRA repository Accession number: PRJNA1137155, http://www.ncbi.nlm.nih.gov/bioproject/1137155.

## 1 Introduction

Accurate analysis of viral genomes is crucial for effectively monitoring and understanding the high genetic diversity and evolution observed in viruses worldwide (Beerenwinkel *et al.* 2012, Posada-Cespedes *et al.* 2017, Kijak *et al.* 2019). Precise reconstruction of these viral genomes is essential to obtain accurate biological representations of individual genomes or quasispecies/variants that might exist (Vasiljevic *et al.* 2021). Traditional Sanger sequencing has been a reliable method for viral genome analysis over the years (Keele *et al.* 2008, Salazar-Gonzalez *et al.* 2009, Baalwa *et al.* 2013). It is however hindered by high costs, limited sequencing depth, and low throughput, making it impractical for large cohort studies that involve large sample numbers (Posada-Cespedes *et al.* 2017, Tovanabutra *et al.* 2019).

To address these limitations, Next-Generation Sequencing (NGS) methods, particularly the Illumina platform, have gained popularity due to their high throughput and increased sequencing depth (Vincent *et al.* 2017, Posada-Cespedes *et al.* 2017, Tovanabutra *et al.* 2019). For commonly used technologies like Illumina, pre-sequencing steps such as genome fragmentation can create challenges in accurately reconstructing repetitive regions. These regions may be incorrectly mapped during the assembly process, leading to potential errors (Klassen and Currie 2012, Schirmer *et al.* 2016). The third-generation sequencing platform [i.e. Nanopore (ONT), and PacBio] offers long reads with high throughput, significantly improving genome coverage. This partially overcomes the limitations of short reads observed in both Sanger and other NGS platforms.

Much as PacBio, it can achieve a remarkable read accuracy of 99.997% (Wenger *et al.* 2019). Its widespread field application is hindered by high costs, including the expense of sequencing instruments and per-sample processing. Additionally, the sequencing workflow is complex, requiring intricate sample preparation and specialized infrastructure. Another major challenge is the substantial computational and data storage demands (Espinosa *et al.* 2024). Long-read sequencing produces large datasets that necessitate high-performance computing resources, adding to the overall complexity and cost of implementation. On the other hand, ONT has demonstrated relatively lower sequencing cost (Leggett and Clark 2017, Wright *et al.* 2021) and can span larger genomic repeats and complex genomic structures (Wright *et al.* 2021). However, ONT platform has been hampered by sequencing errors that result in spurious substitutions, insertions and deletions (indels), and single-nucleotide polymorphisms (SNPs). This can pose a significant problem when accurately reconstructing true biological genetic variation in said organism (Speranskaya *et al.* 2018, Tan *et al.* 2022).

The conventional methods for genome assembly are either reference-guided or *de novo* assembly (Sohn and Nam 2018). *De novo* assembly involves reconstructing genomes from reads without prior knowledge of their sequence or order. This allows for the construction of genomes of novel organisms, thus facilitating the identification of structural variants and complex rearrangements, such as indels and translocations. However, error rates in long reads remain a significant constraint to this approach (Lischer and Shimizu 2017, Sohn and Nam 2018). On the contrary, the reference-guided approach involves aligning query reads to a reference genome of a closely related species. This method involves comparing genetic information between the query reads and the reference genome to construct a draft genome assembly (Lischer and Shimizu 2017). Assemblies generated via this approach may exhibit a bias toward the selected reference, potentially neglecting divergent genomic regions/variations, leading to reduced genomic diversity within the assembly (Wymant *et al.* 2018). Furthermore, inaccuracies present in the reference genome can compromise the assembled draft genomes (Lischer and Shimizu 2017).

**Figure 1.**
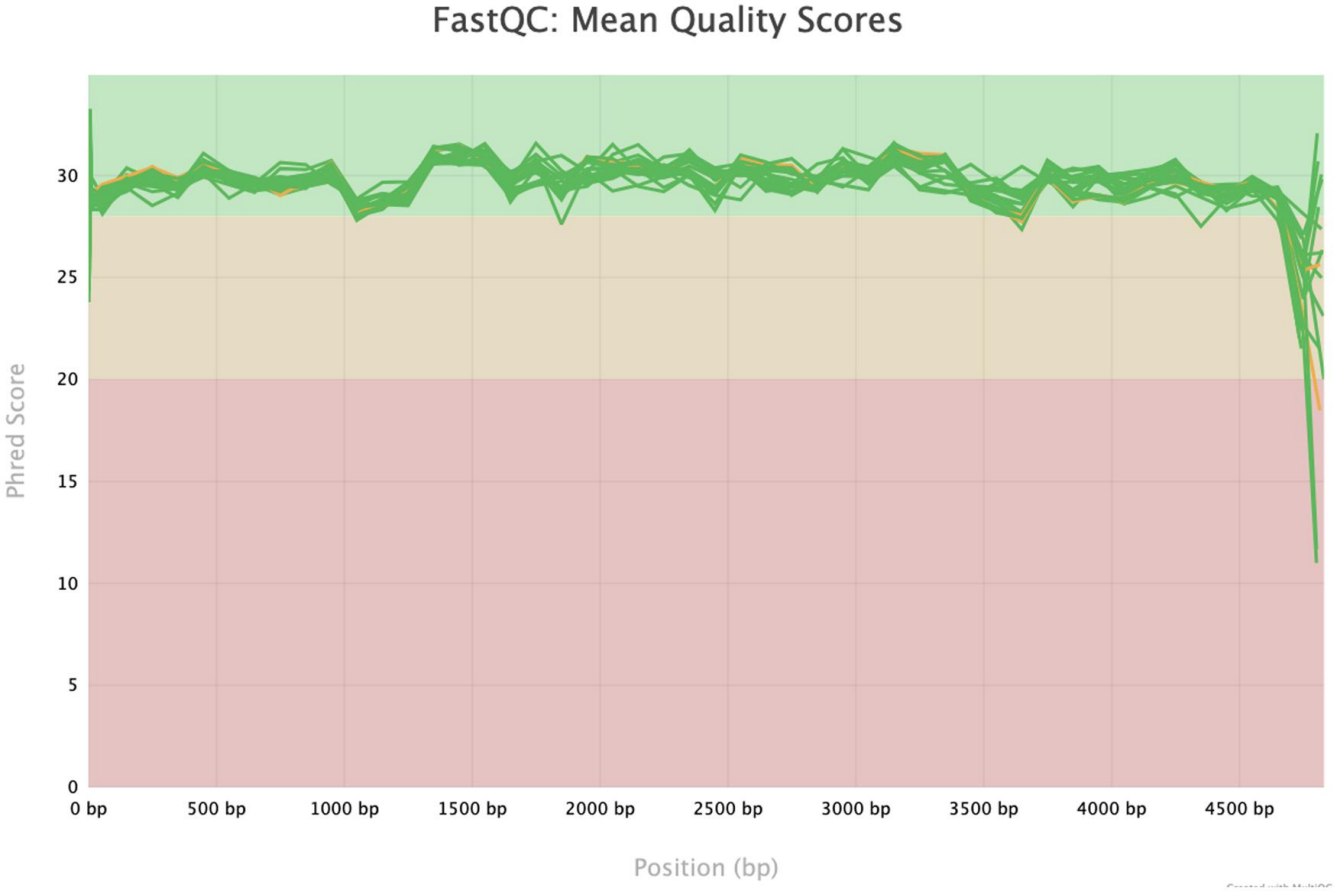
Phred scores of ONT reads sequences using R10 flow-cells and Q20 sequencing kit. The average Phred was 30, which is close to what Illumina produces. The graph was generated using FASTQC and Multiact packages (Ewels *et al.* 2016).

Hybrid assembly tools that combine both short and long reads have significantly improved the accuracy of genome reconstruction from raw reads (Wick *et al.* 2017, De Maio *et al.* 2019). However, this approach is costly and time-consuming, as it requires sequencing of a sample on both long and short-read platforms (Wick *et al.* 2017). However, in response to these errors reported in long-read sequences, ONT has released a new sequencing kit Q20+ and R10 flow cells with improved technology. The comparison of ONT reads generated with R10 flow cells and Q20+ sequencing kit showed that ONT can produce accurate assemblies with a depth of ≥40×. Moreover, the Phred score of reads produced with this R10 and Q20+ sequencing kit showed an increase to 30 (>99% sequence accuracy) ranging from 20 to 34 (Fig. 1) (Bogaerts *et al.* 2024, Hong *et al.* 2024, Lerminiaux *et al.* 2024, Ritchie *et al.* 2024, Sanderson *et al.* 2024).

Additional ONT genome assembly tools for viral sequences have been developed. However, some of these tools (e.g., Genome Detective, NanoHIV, and AccuVIR) (Wright *et al.* 2021) do not perform basecalling from the raw sequence reads, while others such as AccuVIR require error-corrected/assembled reads to generate consensus sequences. In addressing these gaps, we developed the BonoboFlow pipeline tool. BonoboFlow is a Nextflow pipeline designed to facilitate high-accuracy basecalling, error correction, assembly, or haplotype reconstruction. The pipeline also corrects reading frames in draft assemblies, ensuring the output of accurate and reliable viral genomes. The pipeline optimizes critical stages, including read filtering for quality assurance, error correction, draft genome assembly/haplotype reconstruction, and subsequent polishing. Ultimately, BonoboFlow furnishes consensus sequences that authentically reflect the haplotypic diversity present in the sequenced samples while concurrently rectifying any frame shifts, making it suitable in field and viral surveillance settings.

## 2 Materials and methods

### 2.1 Pipeline development

**Figure 2.**
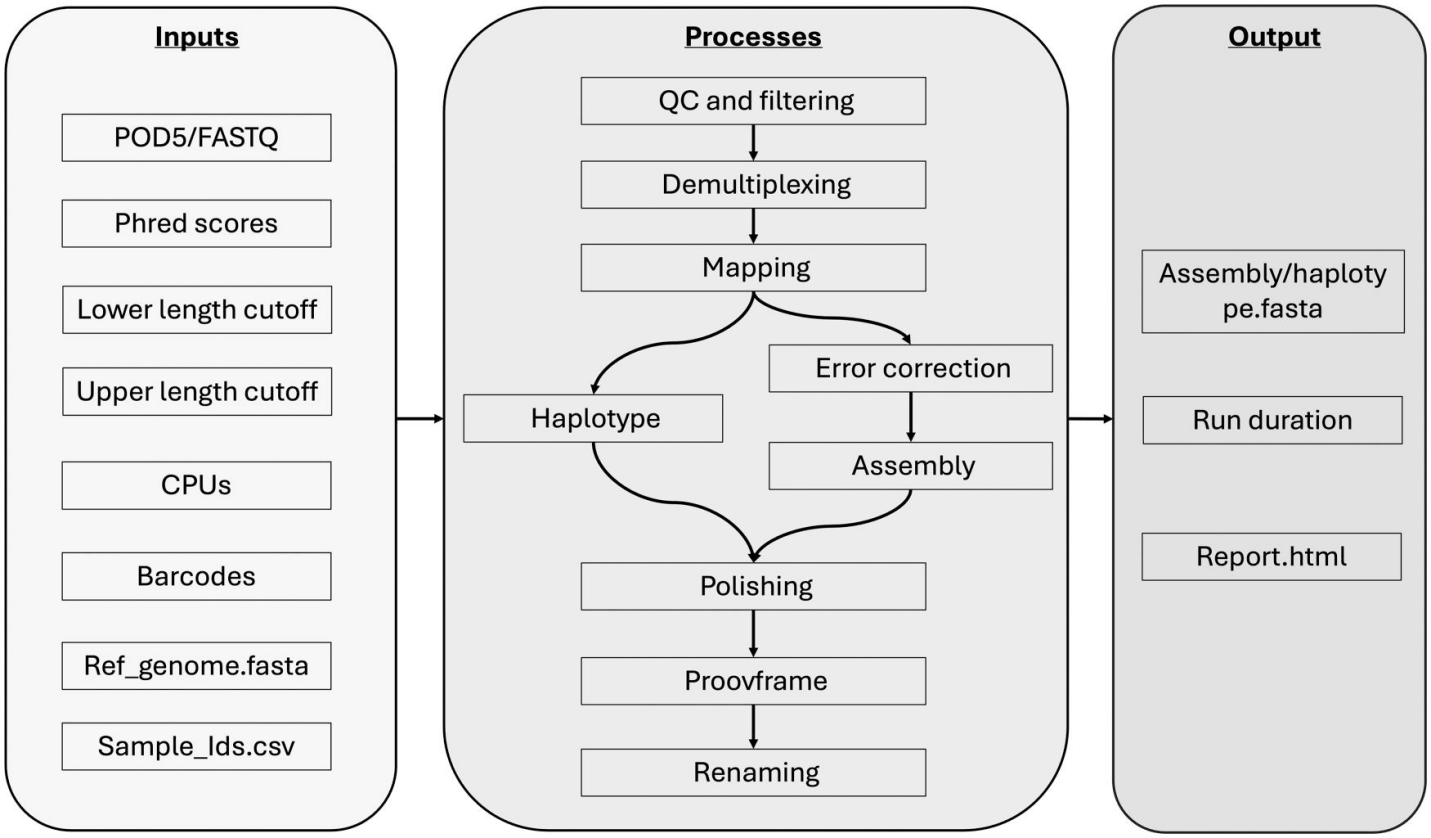
Pipeline flow chart. The pipeline utilizes various steps, including adapter removal, demultiplexing, contamination filtering, error correction, haplotype reconstruction/genome assembly, and consensus sequence polishing. It aims to achieve accurate and reliable reconstruction of viral genomes.

BonoboFlow is a comprehensive pipeline that consists of several key optimized steps **(**Fig. 2) to ensure accurate and reliable assembly/haplotype reconstruction of viral genomes. The pipeline begins by reading from a directory containing raw unpaired POD5/FAST5 or base-called FASTQ reads as input. A Dorado basecaller Version 0.7.2 is incorporated to basecall POD5/FAST5 files with a Super high accurate (SUP) model with simplex basecaller. To eliminate any adapter sequences, chopper version 0.8.0 is employed (De Coster and Rademakers 2023). Subsequently, the trimmed sequences undergo demultiplexing using Dorado version 0.7.2. To enable the accurate identification of individual samples by the allocated barcodes during library preparation, both the front and rear barcodes are required for a read to be called. A long-read aligner, Minimap2 version 2.28-r1209 (Li 2018), and Samtools version 1.2 (Li *et al.* 2009) together with a desired reference in the FASTA format are then used to eliminate host and other contaminating reads, retaining only the desired viral reads. The reads were then error corrected using a haplotype-aware tool (VeChat) to correct erroneous sequences (Luo *et al.* 2022b). These corrections are essential for improving the accuracy of the sequence reads. Depending on the specific requirements (i.e. viral genome construction), the corrected sequence reads are subsequently utilized for either genome assembly using Flye version 2.9.4 (Kolmogorov *et al.* 2020) or haplotype construction using Strainline (Luo *et al.* 2022a) with some modification to enable efficient memory usage. The haplotype construction was optimized using a maximum and local divergence of 0.01. The draft consensus sequences resulting from the assembly process then undergo additional genome polishing steps, using Medaka version 0.11.2 to further refine the accuracy of the obtained consensus sequence by removing any remaining spurious indels and SNPs. Finally, the polished consensus sequence is examined for frameshifts and to ensure reading frame correction using DIAMOND version 2.1.9 and proovframe version 0.9.7 (Buchfink *et al.* 2021, Hackl *et al.* 2021). These steps guarantee the fidelity of the final assembled sequence, providing a reliable representation of the viral genome.

### 2.2 Read simulation across diverse viral genomes

The performance of BonoboFlow was first evaluated using simulated sequencing reads from a range of viral genomes with varying levels of genomic complexity. Simulated sequencing reads were generated using PBSIM2 (Ono *et al.* 2021), a tool for generating long-read simulation data based on real sequencing error profiles. The sequencing reads were simulated at a depth of 100×, using the R103 model. The simulation parameters were set to produce error rates of 10% (90% accuracy).

Read simulations focused on different strains of viruses including Zika, polio, Adenovirus, HIV, and vaccinia viruses (Table 1). Each virus was represented by more than one strain, which were later pooled into a single FASTQ file before downstream analysis against BonoboFlow and each of the benchmarking pipelines.

**Table 1.** Performance of the BonoboFlow against the benchmark pipelines including original Strainline, AccuVIR, and Genome Detective based on the percentage sequence similarity of consensus sequences from the three benchmark pipelines against original sequences used in the read simulations.

Viruses	Accession number	Similarity comparison between different pipeline against the original reference sequences			
		BonoboFlow (%)	Original Strainline	AccuVIR	Genome Detective
**Polio**	PQ812274.1	99.99	100	100	^a^
	PP533216.1	99.97	99.99	^a^	^a^
	PP506139.1	100	100	^a^	52.28
**HIV**	AB253421.1	99.94	99.89	99.94	87.01
	MF373153.1	99.87	99.07	^a^	^a^
	AY772699.1	99.97	100	^a^	^a^
	AB253429.1	99.93	99.54	^a^	^a^
	EF1558043.1	99.59	98.44	^a^	^a^
**Adeno**	NC_012959.1	99.86	^b^	100	100
	PV243660.1	99.67	^b^	^a^	99.22
	PQ16474.1	99.50	^b^	^a^	99.88
	PQ212777.1	99.80	^b^	^a^	^a^
	NC_001454.1	99.42	^b^	^a^	100
**Zika**	KU322639.1	99.34	99.96	99.94	93.94
	AY632535.2	99.99	99.94	^a^	^a^
	KU955591.1	99.98	99.97	^a^	^a^
**Vaccinia**	NC_006998.1	43.23	^b^	92.39	^c^
	NC_004105.1	98.02	^b^	^a^	^c^
	MT_227314.1	99.39	^b^	^a^	^c^
	LT_993228.1	97.77	^b^	^a^	^c^
	KT_184691.1	98.3	^b^	^a^	^c^
	BK_013341.1	98.59	^b^	^a^	^c^
	HQ_420897	32.44	^b^	^a^	^c^

a The pipeline did not produce any haplotype corresponding to the particular input read.

b The pipeline did not complete due to high memory usage.

c The pipeline did not start due to sequences being too long (>100 000 000).

### 2.3 Sequenced datasets description using HIV-1 as a model organism

The long-read sequencing data used in this study was generated on the Oxford Nanopore Technologies (MinION) platform, utilizing the R10 flow cell [FLO-MIN114 (R10.4.1)] and a Q20+ sequencing kit (SQK-LSK114). Sequencing libraries were prepared from HIV-1 3ʹ amplicons, which were amplified from viral RNA extracted from 24 archived HIV-1 plasma samples. These samples were stored at the MRC/UVRI & LSHTM Uganda Research Unit in Uganda. The raw data consisted of long reads with an average read length of ∼4700 base pairs of HIV-1 virus, allowing for the comprehensive coverage of genomic regions and the ability to span complex genomic structures and repetitive elements. The sequencing depth, defined as the average number of times each base in the viral genomes was sequenced, was targeted to achieve a minimum depth of an average of 50× coverage. The data quality was assessed using basecalling scores and other quality metrics provided by the ONT sequencing platform, with an average Phred quality score of 30, indicating high accuracy in basecalling.

#### 2.3.1 HIV-1 RNA extraction and cDNA synthesis

RNA extraction was carried out using QIAamp RNA Mini Kit by Qiagen Inc, Valencia, CA, USA following the manufacturer’s instructions. The recovered RNA was quantified and stored at -80°C before downstream analysis. The cDNA synthesis was carried out using 1.R3.B3R primer (5′-ACTACTTGAAGCACTCAAGGCAAGCTTTATTG-3′) using SuperScript IV (SSIV) reverse transcriptase (Invitrogen). The reaction volume was prepared in two steps. The master mix A contained viral RNA, 10 μM of reverse primer 1.R3.B3R, and 10 mM of deoxynucleotide triphosphate (dNTP). The reaction tube was incubated for 5 min at 65°C to denature secondary RNA structures followed by incubation on ice for 5 min. The Master mix B contained 1× of SSIV RT buffer, 1.5 μl of 100 mM dithiothreitol (DTT), 40 U/μl of the RNaseOUT, 200 U/μl of SuperScript IV Reverse Transcriptase, and Master mix A. This was incubated at 50°C for 30 min. The RT enzyme was heat-inactivated at 70°C for 10 min. The synthesized cDNA was either used immediately or stored at −80°C for future use.

#### 2.3.2 Amplification

Amplification was carried out using a nested PCR. The primary reaction was performed in 25 μl reactions containing 5× Phusion High-Fidelity buffer, 3% DMSO, 2.5 mM of each dNTP, 10 μM of each primer (forward: B3F1 (5′-ACAGCAGTACAAATGGCAGTATT-3′: reverse 1.R3.B3R), 0.02 U/μl Phusion^®^ High-Fidelity DNA Polymerase (New England BioLabs) and 2 μl of template cDNA. The amplification was carried out using 35 cycles at 55°C annealing temperature. The second reaction used 2 μl of the first reaction, B3F3 (5′-TGGAAAGGTGAAGGGGCAGTAGTAATAC-3′), and 2.R3.B6R (5′-CCTTGAGTGCTTCAAGTAGTGTGTGCCCGTCTGT-3ʹ). Primers. The rest of the reaction and cycling conditions were the same as the primary PCR. The expected output product size was around 4.7 kb.

#### 2.3.3 Library preparation and sequencing

Before library preparation, the PCR product was cleaned using the Beckman Coulter™ Agencourt AMPure XP kit in a ratio of 1:1. The clean products were then eluted in 30 μl of elution buffer (EB, QIAGEN). The cleaned products were used for end repair using the NEBNext^®^ Ultra™ II End Repair Module (E7546) and NEBNext Quick Ligation Module. The end prep reaction volume contained 1.75 μl of Ultra II End Prep Reaction buffer, 0.75 μl of End Prep Enzyme, and 100 ng of the purified PCR product. The mixture was incubated at 20°C for 20 min, followed by heat inactivation at 65°C for 5 min. Individual barcodes were added to each of the end-repaired samples. The DNA library was then pooled together and cleaned using the Beckman Coulter™ Agencourt AMPure XP kit. The pooled library was eluted in 30 µl. The adapter mix was added to the library and cleaned using a long fragment buffer. A total of 100 µg of the cleaned adapter-ligated library was loaded onto the MinION flow cell (R10.1) containing 1250 pores. The raw files were analyzed using the newly developed BonoboFlow and benchmarked against AccuVIR and Genome Detective pipelines as described in Section 2.4.

### 2.4 Pipeline benchmarks

The BonoboFlow pipeline was benchmarked against AccuVIR (a viral genome assembly and polishing tool that intakes error-prone long reads such as ONT reads; https://github.com/rainyrubyzhou/AccuVIR) (Yu *et al.* 2023), original Strainline (an approach to assemble viral haplotypes from noisy long reads without a reference sequence) (Luo *et al.* 2022), and Genome Detective (a bioinformatics application for the analysis of microbial molecular sequence data) (Vilsker *et al.* 2019) using both the sequenced HIV-1 and simulated viral reads. Since neither of the benchmarking tools can be able to process POD5, which was the output format from the ONT device, read processing was carried out using Dorado to baseball with super accuracy and demultiplexed into individual barcodes. In addition to this, reads that were used for AccuVIR were further assembled by Flye (Kolmogorov *et al.* 2019, 2020, Lin *et al.* 2016).

#### 2.4.1 Performance comparison

The performance comparison of BonoboFlow was carried out in two fold; firstly, consensus sequences from real HIV-1 dataset derived from BonoboFlow, AccuVIR, and Genome Detective were aligned using Multiple Alignment using Fast Fourier Transform (MAFFT) with default settings (Katoh and Standley 2013). The multiple sequence alignment (MSA) files were used to construct a Maximum likelihood tree with 1000 bootstrap replicates and a GTR+F + I+R2 model in IQ-TREE 2 (Minh *et al.* 2020). The consensus tree file was visualized using the Figtree package.

For the simulated reads, consensus sequences generated by each pipeline were aligned to the reference strains used in the simulation. The percentage identity between each consensus sequence and its corresponding reference strain was calculated to evaluate the performance of each pipeline.

## 3 Results

### 3.1 BonoboFlow performance on simulated data compared to original Strainline, AccuVIR, and Genome Detective

The pairwise alignment of the reconstructed sequences from different pipelines against the references showed that all pipelines performed well with alignment percentages typically above 99%, especially Polio, HIV, Adenovirus, and Zika viruses. BonoboFlow, Strainline, and AccuVIR often produced highly similar reconstructions, particularly for Polio and Zika genomes. However, a notable difference between pipelines was their ability to generate outputs across different strains that were used in data simulation. In several cases, some pipelines (especially AccuVIR and Genome Detective) did not produce all the strains used in simulation rather generated one consensus sequence. The original Strainline on the other hand was limited on high memory usage resulting in pipeline abortion especially when presented with a big dataset or long genomes like adenovirus and vaccinia (Table 1).

### 3.2 Benchmarking BonoboFlow against Genome Detective and AccuVIR using real HIV-1 datasets

**Figure 3.**
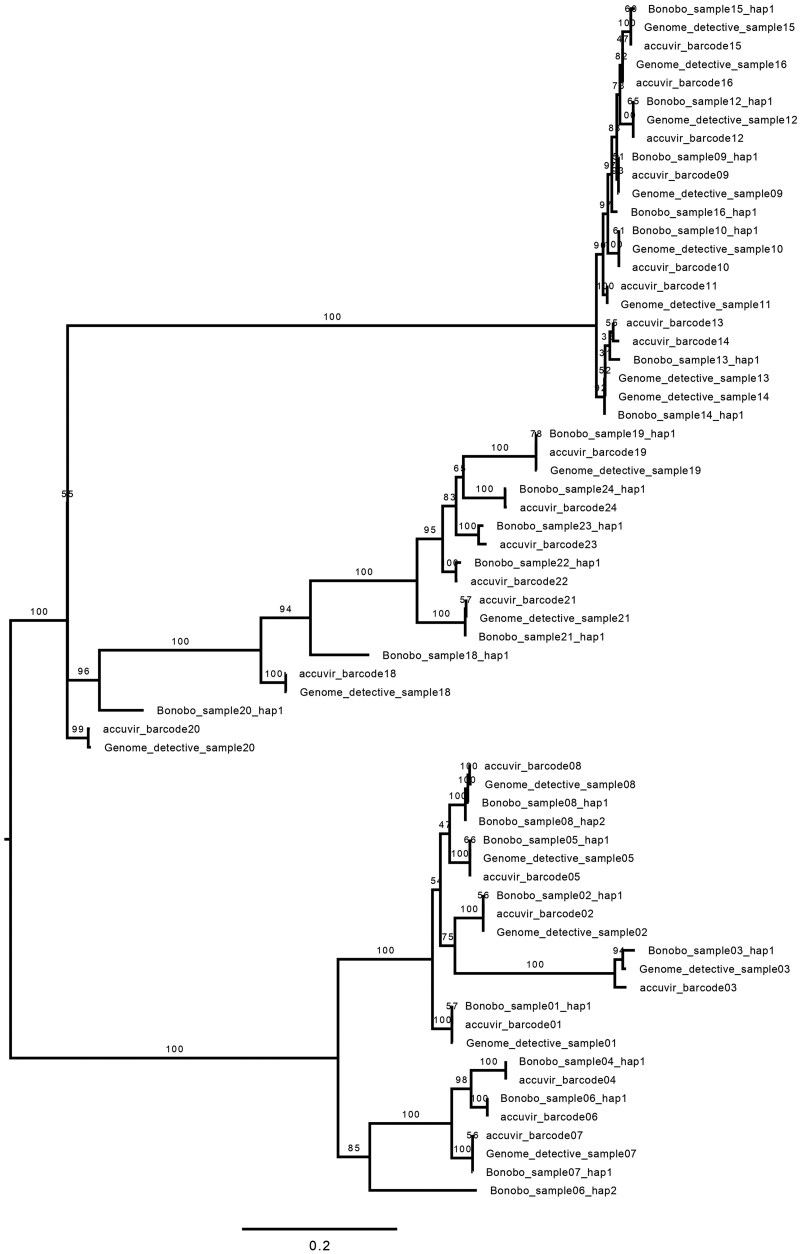
Phylogenetic tree of the consensus sequences from the three platforms (i.e. BonoboFlow, AccuVIR, and Genome Detective). The tree identified that sequences from the samples that were assembled from different pipelines clustered together. The tree was constructed using IQ-TREE 2 and rendered using FigTree.

Phylogenetic analysis (Fig. 3) indicated that all the pipelines generated consensus sequences that clustered together highlighting a high level of agreement. The percentage sequence similarity of the consensus sequence generated from BonoboFlow against AccuVIR and Genome Detective ranged from 75.7% to 100% (Fig. 3 and Table 2). The mean and median sequence similarities against the two-benchmarking pipeline were 95.9% and 99.9% and 96.19% and 99.9% for AccuVIR and Genome Detective, respectively.

**Table 2.** Percentage sequence similarity of consensus sequences from the two benchmarking pipelines.

Bonobo	Percentage similarity	
	AccuVIR	Genome_Detective
Bonobo_sample_01_hap1	99.9	99.9
Bonobo_sample_02_hap1	75.7	75.7
Bonobo_sample_02_hap2	100	100
Bonobo_sample_03_hap1	89.2	74.9
Bonobo_sample_03_hap2	83.8	99.8
Bonobo_sample_05_hap1	98.2	98.1
Bonobo_sample_06_hap1	99.9	99.9
Bonobo_sample_07_hap1	100	^a^
Bonobo_sample_09_hap1	100	100
Bonobo_sample_10_hap1	100	100
Bonobo_sample_11_hap1	100	100
Bonobo_sample_12_hap1	100	100
Bonobo_sample_13_hap1	100	100
Bonobo_sample_14_hap1	100	100
Bonobo_sample_15_hap1	99.9	100
Bonobo_sample_16_hap1	98	98
Bonobo_sample_18_hap1	88.1	^a^
Bonobo_sample_18_hap2	89.7	^a^
Bonobo_sample_19_hap1	100	99.8
Bonobo_sample_20_hap1	89	90.1
Bonobo_sample_20_hap2	86.9	87.8
Bonobo_sample_21_hap1	100	^a^
Bonobo_sample_22_hap1	99.6	^a^
Bonobo_sample_23_hap1	99.9	99.9
Bonobo_sample_24_hap1	99.8	99.8

a Consensus sequences were not produced.

Closer observation with multiple sequence alignments elucidated the spectrum of genetic variation captured by each benchmarking pipeline. These sequence variations were observed especially in those samples which were indicated to have more than one haplotype by BonoboFlow. Specifically, a few BonoboFlow-derived sequences displayed a dichotomy wherein certain variants closely mirrored the consensus sequence with minimal mutational divergence, while others exhibited pronounced genetic disparities.

### 3.3 Runtime analysis

To evaluate the computational efficiency of BonoboFlow, we conducted experiments on a Scientific Linux 7.5 computing system comprising 6 Compute Nodes, 192 CPU cores, and 1.5 TB of memory. This was carried out using a 2.1 GB FASTQ file containing 772 231 reads of HIV-1 reads that were sequenced in this study (N50 4500 bp) (SRA Accession number: PRJNA1137155, http://www.ncbi.nlm.nih.gov/bioproject/1137155). The results showed that BonoboFlow completed in 1 h, 3 min, and 21 s, processing data from basecalled FASTQ reads to the final consensus FASTA sequences. Notably, the haplotype construction step accounted for a significant portion of the total runtime compared to other processes.

## 4 Discussion

The performance analysis of BonoboFlow highlights its capability to assemble viral genomes across a range of complexity levels and sequencing error profiles. In addition, it provides an end-to-end Nanopore read processing from raw POD5/FAST5 to consensus genomes. By evaluating its performance on viruses spanning small (e.g. Polio virus) to highly complex genomes (e.g. Vaccinia), we assessed its capability in different genomic contexts and its ability to correctly produce haplotypes.

Using simulated reads generated with PBSIM-2 (Ono *et al.* 2021) at 10% error rates (90% accuracy) and a sequence depth of 100×, we generated long reads resembling ONT reads using different viral strains. BonoboFlow performed well in generating consensus sequences with a sequence similarity to the original strains used in simulation with a percentage local similarity hitting 100% in some strains (Table 2). However, performance declined when assembling larger and more complex genomes like vaccinia. This reduction is likely due to challenges associated with assembling long genomes, including the presence of repetitive regions, in addition to high computational demands. Previous studies have noted similar difficulties in assembling highly diverse viral populations, where genetic variation complicates genome reconstruction. Additionally, the presence of virus–virus chimeras and low viral sequence coverage may contribute to incomplete or fragmented assemblies (Deng *et al.* 2021, Dovrolis *et al.* 2021, Yang *et al.* 2012). On the other hand, benchmarking tools such as original Strainline also achieved a high recovery of the consensus sequences against the original reads, except in few circumstances where, it failed to run to completion due to high memory usage especially when presented with large dataset.

When tested on HIV-1 amplicon-based sequenced on Mk1C device, BonoboFlow was able to identify more than one haplotype in some sample. This is particularly relevant for RNA viruses, which exhibit high mutation rates and genetic diversity. Accurate haplotype reconstruction is essential for understanding viral evolution. Similar tools, such as RVHaplo, HaploDMF, and VirStrain (Cai and Sun 2022, Cai *et al.* 2022, Liao *et al.* 2022), have also emphasized the importance of reconstructing viral haplotypes and strain compositions. When benchmarked with previous tools using real HIV-1 dataset, BonoboFlow produced consensus sequences with strong similarity to those generated by AccuVIR and Genome Detective. Sequence similarity between BonoboFlow against AccuVIR and Genome Detective showed mean and median values of 95.9% and 99.9% and 96.19% and 99.9%, respectively. Phylogenetic analysis further supported these findings, showing that consensus sequences from all three platforms clustered closely together.

BonoboFlow integrates multiple optimized and harmonized tools for viral genome assemblies, from sequenced raw reads to the final consensus sequence (Fig. 2). While most of the alternative available tools require preprocessed input data such as basecalling, demultiplexing, and error collection, previous studies have emphasized the importance of optimizing computational tools for genome assembly using ONT data. For instance, Senol Cali *et al.* (2019) conducted a comprehensive analysis of Nanopore sequencing technology and associated tools, identifying current bottlenecks and proposing future directions for improvement (Senol Cali *et al.* 2019). Further, Sutton *et al.* (2020) explored strategies for optimizing experimental design in genome sequencing and assembly with ONT, highlighting the need for tailored computational approaches to handle the unique challenges posed by Nanopore data (Sutton *et al.* 2020). These studies underscore the necessity of developing and refining computational pipelines to enhance the accuracy and efficiency of ONT-based genome assemblies while minimizing errors.

As an open-source tool available on GitHub (https://github.com/nchis09/Bonobo), BonoboFlow is accessible and can handle larger datasets more efficiently compared to online tools, which usually have limitations on input size. In terms of computational efficiency, BonoboFlow processed a 2.1GB file containing 772 231 HIV-1 FASTQ reads of ∼4500 bp (N50) in just over an hour. The most time-intensive step was haplotype construction, a necessary process for resolving multiple viral variants in complex samples. Despite this, the overall runtime was reasonable given the scale and complexity of the dataset. BonoboFlow also demonstrated flexibility by performing well on high-performance Linux computing clusters and consumer-grade systems (MacOS and Ubuntu).

While BonoboFlow successfully assembled viral genomes across various conditions, some areas warrant further refinement. Although it performed well even under higher sequencing error rates (10%), current ONT R10 & Q20+ chemistry offers read accuracy of approximately 99% (Cuber *et al.* 2023, Zhang *et al.* 2023), suggesting that BonoboFlow can perform even much better while running sequences from this technology in field applications. Nonetheless, future improvements could also enhance variant calling, particularly in extra-long genomes with high level of genetic repeats. Overall, BonoboFlow provides a useful approach for viral genome analysis, balancing accuracy, efficiency, and accessibility. With continued development, it has the potential to improve the analysis of viral genomes in research areas spanning human health, veterinary medicine, and environmental surveillance.

## 5 Possible applications of BonoboFlow

BonoboFlow can be used in different aspects of research, such as virus surveillance of new or circulating variants/viruses. It can also be used in phylogenetic studies that aim at analyzing population clusters of viral genomes that are generated using MinION sequencing. Consensus sequences generated from BonoboFlow can further be used to carry out multiple sequence alignment, phylogenetic tree, open reading frames estimation, and subtyping/genotyping among others for viral genome analysis/characterization.

## 6 Conclusion

The BonoboFlow pipeline showed a notable level of agreement with AccuVIR and Genome Detective, with mean and median sequence similarities of 95.9% 99.9%, 96.19%, and 99.9%, respectively. This suggests it can provide reasonably accurate results. However, BonoboFlow had difficulty resolving consensus sequences with long simulated reads, indicating a need for further optimization in this area. As an open-source tool, it remains accessible and adaptable for various research applications. With advancements in sequencing technologies, BonoboFlow has the potential to be a useful option for viral genome analysis, including applications in veterinary and human health research, as well as environmental surveillance for pandemic preparedness.
